# Developing natural products as potential anti-biofilm agents

**DOI:** 10.1186/s13020-019-0232-2

**Published:** 2019-03-20

**Authors:** Lan Lu, Wei Hu, Zeru Tian, Dandan Yuan, Guojuan Yi, Yangyang Zhou, Qiang Cheng, Jie Zhu, Mingxing Li

**Affiliations:** 1 Sichuan Industrial Institute of Antibiotics, Chengdu University, Chengdu, Sichuan People’s Republic of China; 2 Laboratory of Molecular Pharmacology, Department of Pharmacology, School of Pharmacy, Southwest Medical University, Luzhou, Sichuan People’s Republic of China; 3 South Sichuan Institute of Translational Medicine, Luzhou, Sichuan People’s Republic of China; 4 Department of Gastroenterology, Shenzhen Hospital, Southern Medical University, Shenzhen, Guangdong People’s Republic of China; 5 Department of Anaesthesia and Intensive Care, The Chinese University of Hong Kong, Hong Kong, People’s Republic of China; 6 School of Biomedical Sciences, Faculty of Medicine, The Chinese University of Hong Kong, Hong Kong, People’s Republic of China; 7 Department of Internal Oncology, Shandong Cancer Hospital and Institute, Shandong Academy of Medical Sciences, Jinan, Shandong People’s Republic of China

**Keywords:** Anti-biofilm agents, Natural products, QS inhibition, Biofilm-associated infections

## Abstract

Biofilm is a natural form of bacterial growth ubiquitously in environmental niches. The biofilm formation results in increased resistance to negative environmental influences including resistance to antibiotics and antimicrobial agents. Quorum sensing (QS) is cell-to-cell communication mechanism, which plays an important role in biofilm development and balances the environment when the bacteria density becomes high. Due to the prominent points of biofilms implicated in infectious disease and the spread of multi-drug resistance, it is urgent to discover new antibacterial agents that can regulate biofilm formation and development. Accumulated evidences demonstrated that natural products from plants had antimicrobial and chemo-preventive properties in modulation of biofilm formation in the last two decades. This review will summarize recent studies on the discovery of natural anti-biofilm agents from plants with clear-cut mechanisms or identified molecular addresses, as well as some herbs with unknown mechanisms or unidentified bioactive ingredients. We also focus on the progression of techniques on the extraction and identification of natural anti-biofilm substances. Besides, anti-biofilm therapeutics undergoing clinical trials are discussed. These newly discovered natural anti-biofilm agents are promising candidates which could provide novel strategies for biofilm-associated infections.

## Introduction

Billions of years of selective pressures have given rise to numerous strategies in bacteria survival, which adapts this organism to almost any environmental niches. One of preferred growth states for bacteria is known as biofilm which exists in more than 90% of bacteria. Biofilms are multicellular surface-attached communities of bacteria embedded in extracellular matrix (ECM). Quorum sensing (QS), a cell-to-cell communication, has been identified to play critical roles in formation of biofilm with its surrounding ECM. Bacteria living in biofilms show a highly elevated pattern of adaptive resistance to antibiotics and other disinfectants compared to their planktonic compartments. Adaptive antibiotic resistance on the rise globally acts as an obstacle when treating biofilm-associated acute and chronic infections [1–3], such as nosocomial pneumonia cases, surgical wound infections, catheter-associated infections, burn wound infections, ventilator-associated pneumonia, etc. Biofilm-forming has thus caused a large number of problems in health care, food industry, and other fields [4]. On the other hand, the misuse of antibiotics also contributed to development of drug resistance, which might aggravate the bacteria infected disease. Thus, novel strategies other than antibiotics should be developed to combat the bacterial and biofilm formation. In last two decades, novel approaches in preventing biofilm formation and QS have been widely developed and reported including natural products from plants. Many plant natural products have been demonstrated antimicrobial and chemo-preventive properties [5]. It is well known that herbal remedies are employed by different human cultures for centuries and some of those natural products are essential for prevention and treatment for infectious diseases. For example, traditional Chinese medicinal herbs were commonly used in bacterial infection and prevention and some herbs such as *Scutellara*, *Taraxacum* and *Tussilago* exhibited antibacterial ability [6]. Recently, extracts from plants were also reported to regulate biofilm formation and inhibit QS [7]. Regarding that thousands of herbs existed in worldwide and the traditional medicinal herbs have a long history in treated infectious disease especially in China, medicinal herbs might be rich sources and would be more promising for extraction of new products for fighting against biofilm.

In this review, we briefly summarize the mechanisms of biofilm formation and quorum sensing, as well as recent advances in discovery and identification of plant-derived natural products as anti-biofilm agents and extraction approaches for identification of potential components. Besides, plant-derived anti-biofilm therapeutics related clinical trials were also listed and discussed. These newly discovered natural anti-biofilm agents are promising candidates which could provide novel strategies for combating pathogenic bacteria and treatment of biofilm-associated infections.

## Biofilm development and its relation to quorum sensing

**Fig. 1:**
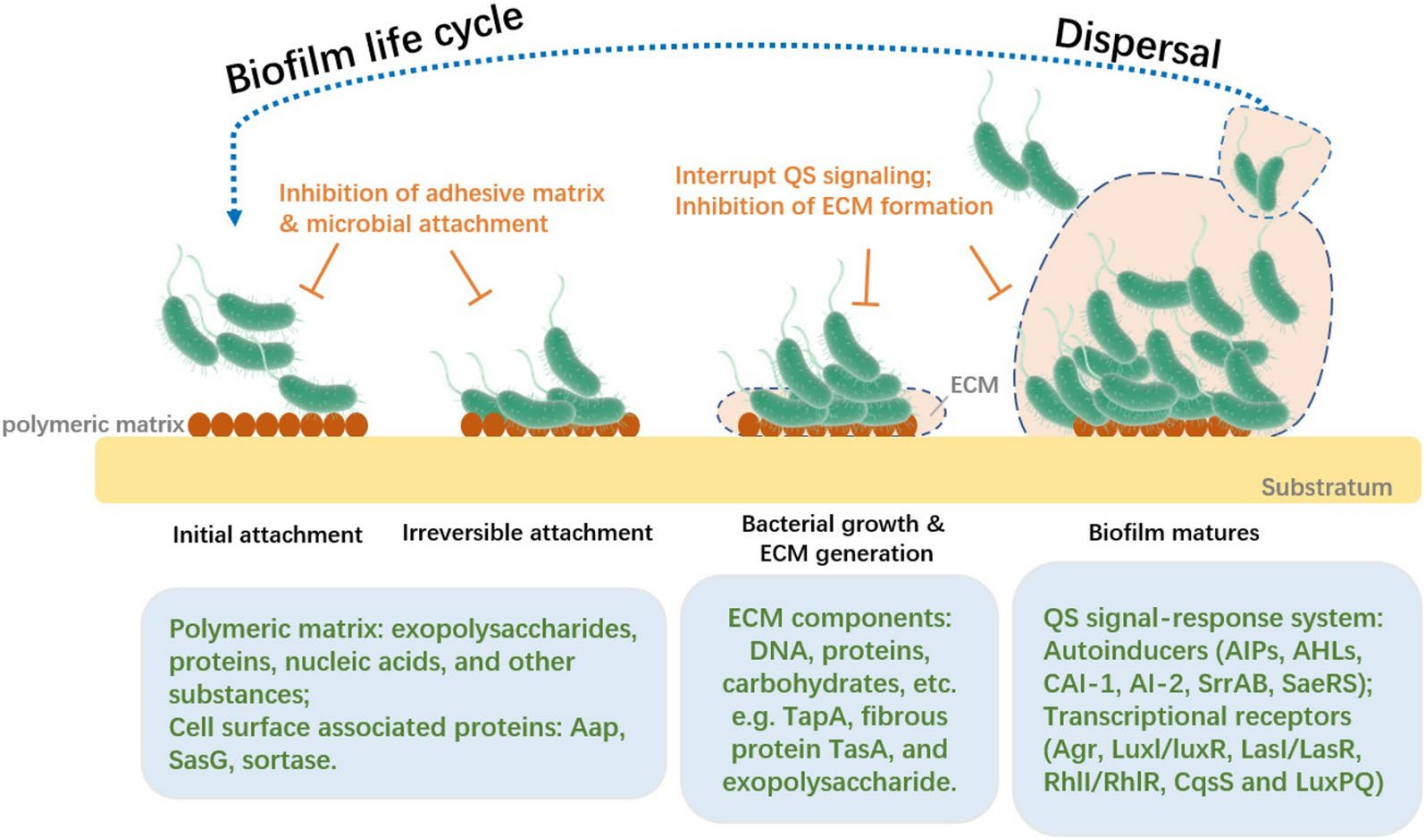
Biofilm formation and quorum sensing. This figure shows four steps in a biofilm life cycle and the associated factors participating biofilm development and quorum sensing. Biofilm starts from bacteria initial attachment, and then develops into irreversible attachment. During these two periods, extracellular DNA, proteases, cell surface proteins or biofilm-associated proteins are involved in biofilm initiation. In the following steps, ECM is generated, and biofilm becomes mature. A cell–cell communication mechanism, called quorum sensing (QS), plays an important role in last two steps of biofilm formation. Multiple autoinducers and corresponding transcriptional receptors are regulating varieties of virulence factors production, contributing to the biofilm control and environmental equilibrium. Last but not least, the bacteria could disperse from the biofilm to find new niches and initiate new biofilm formation, thus resulting in the completion of biofilm life cycle. The strategies on anti-biofilm formation mainly target on each step of biofilm development, including the inhibition of adhesive matrix and microbial attachment, disturbing ECM generation and interruption of QS signaling

Biofilm formation could be defined as one of leading causes for bacteria developing multi-drug resistance. One biofilm life cycle contains four stages, the initial attachment of bacteria, microbial colonies formation, bacterial growth and ECM generation and biofilm matures as the latest stage, followed by the dispersal of the bacteria to find new niches (Fig. 1). The surface of the substratum presents host polymeric matrix, which is mainly composed of exopolysaccharides, proteins, nucleic acids, and other substances, facilitating irreversible attachment of the bacteria. It was reported that cell surface-associated proteins such as Aap and SasG were involved in *Staphylococcus epidermidis* initiating attachment and Aap protein contains G5 domain, which was responsible for bacterial intercellular cell adhesion [8]. Extracellular components, including the surface-exposed protein, the extracellular glucan-binding protein and the glycosyltransferases (GtfE, GtfG and GtfH), also play an important role in cell adhesive abilities [9]. Sortase A (SrtA), a transpeptidase that can anchor cell surface proteins, also elicits extracellular localization and biofilm formation during infection of Gram-positive bacteria, such as *Staphylococcus aureus* [10]. Thus, inhibitors against these adhesion-associated proteins were widely developed and might potentiate a good capacity of anti-biofilm and anti-microbial activities. Then, the adhesive bacteria proliferated into microcolonies. When the biofilm formation became mature, a complex architecture of matrix was formed with water channels for influx of nutrients and efflux of wastes [11]. ECM components contain DNA, proteins, carbohydrates, etc. For example, TapA, fibrous protein TasA, and exopolysaccharide are important components for *Bacillus subtilis* biofilm formation, and spermidine was also essential for activating expression of these matrix components [12]. Different conditions, such as oxygen availability or pH value, in biofilm contributed to different gene expression profiles [13]. Decreased oxygen concentrations within biofilm could lead to increased programmed cell lysis (PCL) and promoted biofilm formation in *S. aureus* [14]. This progression was due to SrrAB and SaeRS-dependent upregulation of AtlA murein hydrolase, followed by release of cytosolic DNA [15]. In addition, there were some studies on genome-wide analysis on biofilm formation and finding some genes associated with biofilm formation such as genes for ClpYQ protease and purine biosynthesis [16]. After the biofilm becomes mature, the bacteria could escape from the biofilm and can initiate new attachment, contributing a new biofilm life cycle.

**Fig. 2:**
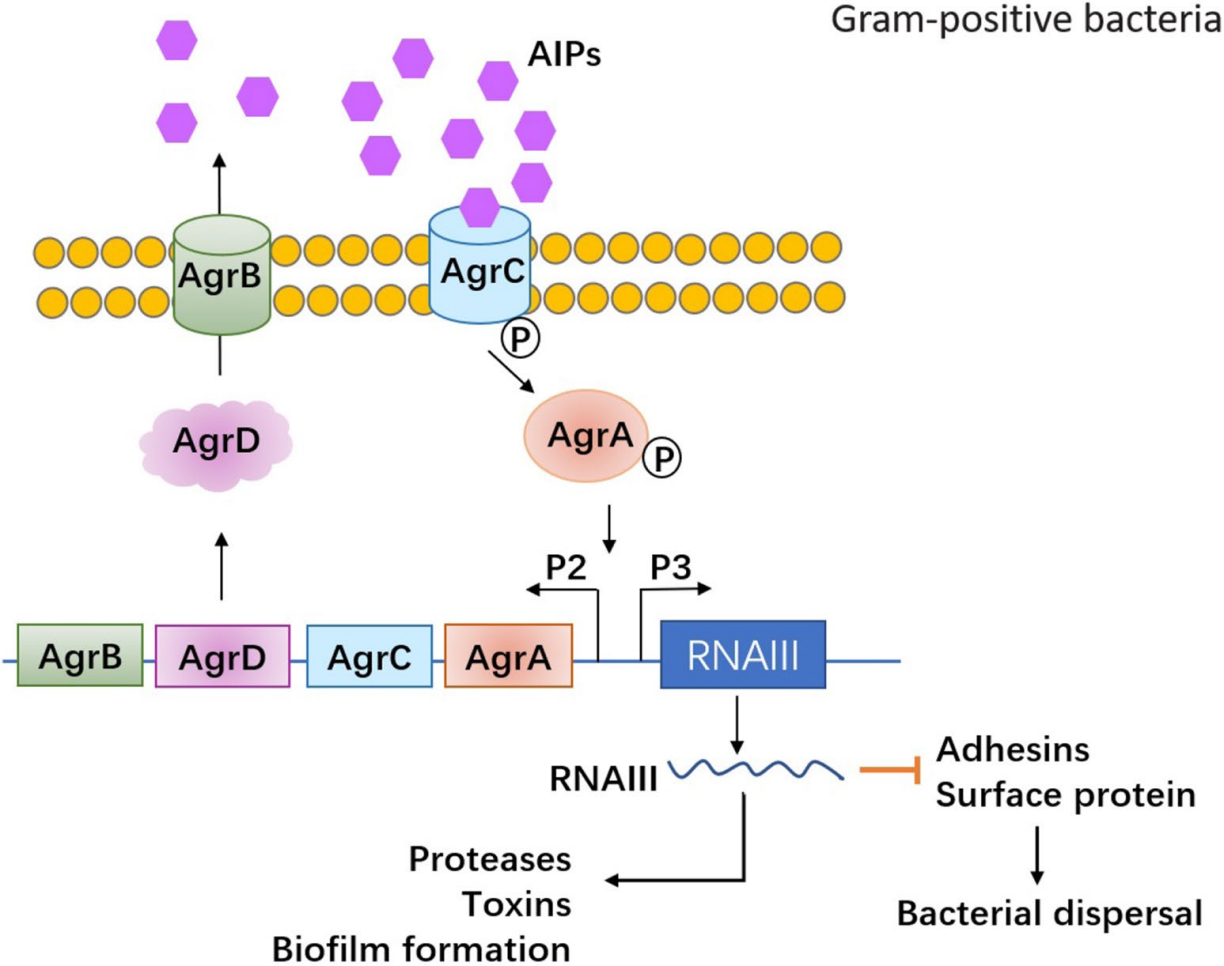
The canonical QS signaling in Gram-positive bacteria and its role in biofilm formation. Agr system was identified as the most classical QS system in Gram-positive bacteria. Agr system in *S. aureus*, the most common bacteria of Gram-positive bacteria, was well investigated, which are important and responsible for production of virulence factors including toxins and proteases. Agr system is controlled by Agr operon, which includes four elements AgrA, AgrB, AgrC and AgrD. AgrD was the precursor of autoinducer peptides (AIPs), the specific autoinducer in Gram-positive bacteria. AgrD is modified by AgrB and secreted to the extracellular matrix. When the bacteria density become high, AIPs will activate the transmembrane protein AgrC. The phosphorylated AgrC further activates AgrA, finally promoting the target genes expression. There are two promoters which can be regulated by AgrA. One is P2 regulating the Agr proteins and another is P3 which can activate RNAIII expression. RNAIII is the key regulator to modulate the expression of QS-related factors and proteins related to biofilm formation. RNAIII can induce upregulation of virulence factors expression such as proteases, toxins and degradative enzymes. On the other hand, RNAIII can also inhibited the expression of cell adhesive proteins and surface proteins, which might contribute to the bacterial dispersal. These dual functional role of Agr system might balance the bacterial swarming and infection. This will also provide therapeutic targets to develop antibiofilm agents, e.g. targeting AIPs, Agrs or RNAIII

**Fig. 3:**
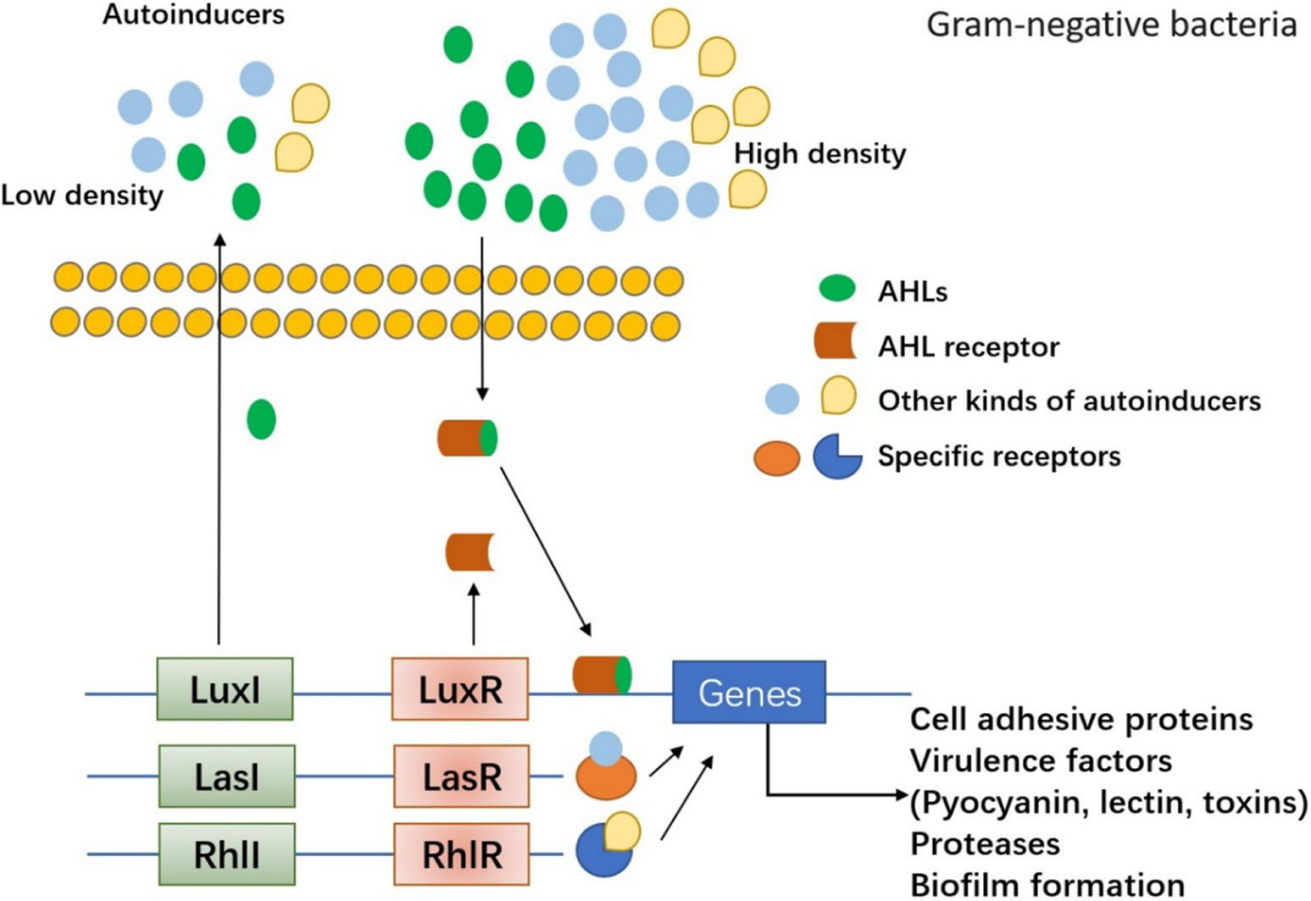
QS signaling in Gram-negative bacteria and its role in biofilm formation. Autoinducer acylhomoserine lactones (AHLs) were commonly produced in Gram-negative bacteria communication and activated corresponding cytoplasmic receptors to modulate targeted genes expression. The canonical key regulators of QS system in Gram-negative bacteria is Luxl/luxR transcriptional factors, which could be activated by AHLs and therefore promoted target genes expression including virulence factors expression such as pyocyanin, lectin, elastase, proteases, toxin and so on. There are also other types of autoinducers (*Pseudomonas* quinolone signal (PQS), CAI-1, AI-2, etc.) and corresponding QS receptors (LasI/LasR, RhlI/RhlR, CqsS and LuxPQ, etc.) varied in different kinds of Gram-negative bacteria. The activated receptors by specific autoinducers finally promote genes expression such as adhesins and virulence factors, which are further involved in biofilm development

The cell-to-cell communication mechanism, quorum sensing (QS), has been found to play a critical role in biofilm formation in both Gram-negative and -positive species. The mechanism underlying the role of QS in biofilm formation has been widely studied. QS enables the bacteria to recognize the population density by sensing and measuring the accumulation of specific self-produced signal molecules secreted by the community [17, 18]. Meanwhile, it alters bacterial gene expression and activates cooperative responses by activating signaling pathways when the population density is high enough to induce the level of accumulated signals in the environment [19]. These genes encode an arsenal of virulence factors, such as exoenzymes, proteases, elastases and pyocyanine, etc. Molecular mechanism involved in QS was widely investigated but was different between Gram-positive bacteria and Gram-negative bacteria, which has been summarized in detail [20–22]. Gram-positive bacteria secreted autoinducer peptides (AIPs) in the environment. As the concentration of AIPs became high, it would bind to the kinase receptors on the bacteria membrane to transmit signal to corresponding transcriptional elements, finally activating related genes expression such as accessory gene regulator (Agr) and RNAIII. Agr system was identified as the most classical QS system in Gram-positive bacteria (Fig. 2). Agr system in *S. aureus,* the most common bacteria of Gram-positive bacteria, was well investigated, which are important and resulting in production of virulence factors including toxins (phenol-soluble modulins PSMs, alpha-toxin, delta-toxin (hld), etc.) and degradative exoenzymes (proteases SspA, SspB, Spl, etc.) [21]. On the other hand, autoinducer acylhomoserine lactones (AHLs) were commonly produced in Gram-negative bacteria communication and bound to cytoplasmic receptors to modulate targeted genes expression when the concentration of AHLs autoinducers in bacteria community became high. The canonical QS system in Gram-negative bacteria is Luxl/luxR transcriptional factors, which could be activated by AHLs and therefore influenced virulence factors production such as pyocyanin, lectin, elastase, proteases, toxin and so on (Fig. 3). There were also other types of autoinducers (*Pseudomonas* quinolone signal (PQS), CAI-1, AI-2, etc.) and associated gens/QS receptors (LasI/LasR, RhlI/RhlR, CqsS and LuxPQ, etc.) varied in different kinds of Gram-negative bacteria [20]. What is more, QS has been shown to influence the biofilm architecture and provide an inherent protection from external factors, such as host immunity and antibiotic therapy [4]. The biofilm life cycle along with the main participators in the process is displayed in Fig. 1. The QS regulator systems in Gram-positive bacteria and Gram-negative bacteria are shown respectively in Figs. 2 and 3. Actively studying the complex state of biofilms and the cellular communication mechanism provides new strategies and targets for scientists to identify QS inhibitors (QSI) and novel therapeutics against biofilm-associated infections.

## Natural anti-biofilm agents with clear-cut mechanisms or identified molecular addresses

**Fig. 4:**
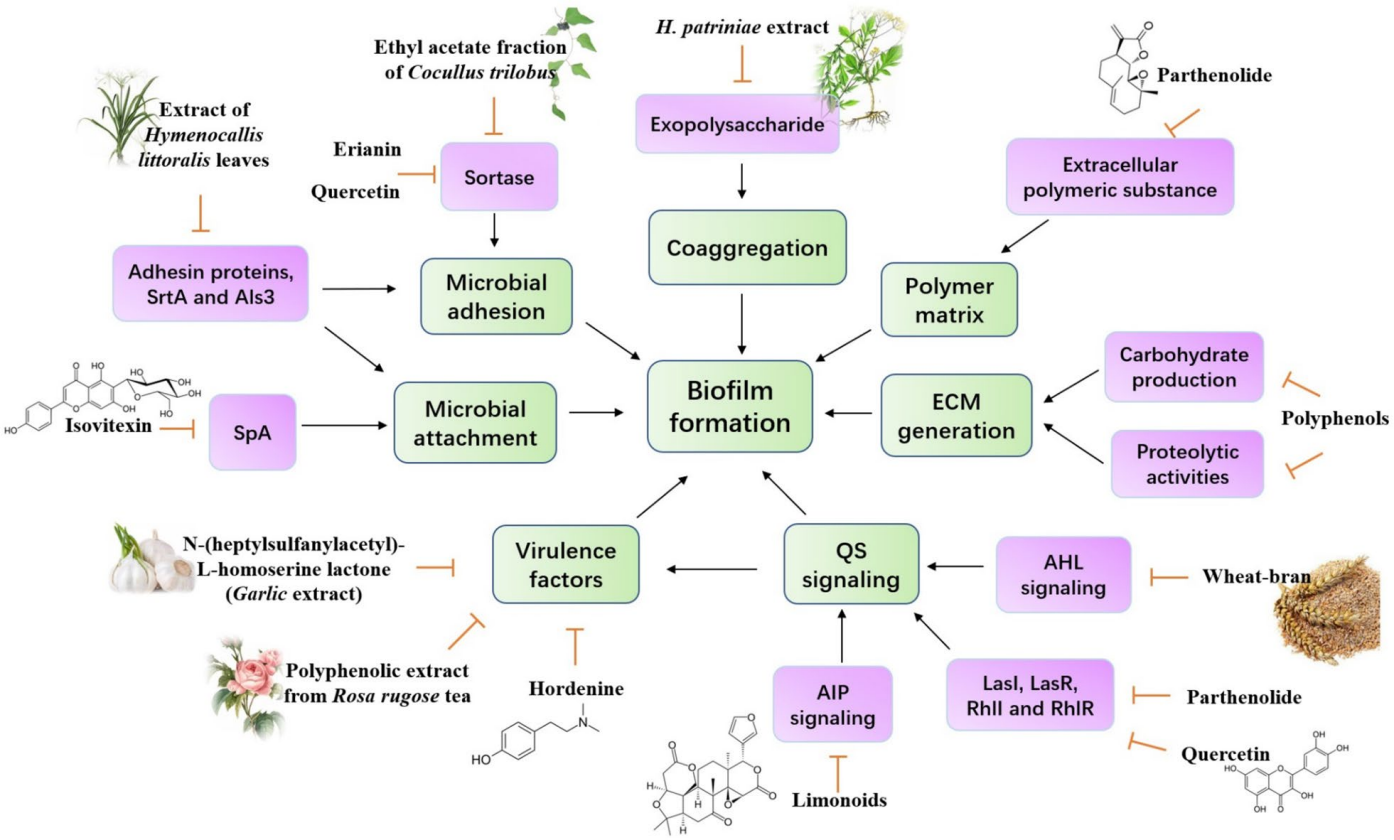
Anti-biofilm agents derived from natural plants and its potential mechanisms. The inhibition of biofilm formation mainly due to serval aspects including the suppression of microbial adhesion and attachment, the inhibition of polymer matrix and ECM generation and interference with bacterial coaggregation and QS network. QS, quorum sensing; SrtA, sortase A; SpA, Staphylococcal protein A; ECM, extracellular matrix; AIP, autoinducer peptide; AHL, autoinducer acylhomoserine lactone

Many plant-derived natural products possessed antimicrobial and anti-biofilm functions in vitro. A variety of molecules derived from natural plants or medicinal herbs extract as well as the underlying mechanisms in anti-biofilm function were identified. The anti-biofilm effects of natural products are mainly relying on the following aspects, the inhibition of formation of polymer matrix, suppression of cell adhesion and attachment, interrupting ECM generation and decreasing virulence factors production, thereby blocking QS network and biofilm development. In the following part, these anti-biofilm agents extracted from medicinal plants, such as garlic, *Cocculus trilobus*, *Coptis chinensis* and so on, were summarized, discussed and was listed in Table 1. We also illustrated the underlying mechanism associated the anti-biofilm effects of these natural products or plants extract in Fig. 4.

**Table 1:** Natural anti-biofilm agents and their molecular mechanisms in anti-biofilm effects

Plants extract/compounds	Mechanism/molecular addresses	Target bacteria	Anti-biofilm effects	References
*N*-(Heptylsulfanylacetyl)-l-homoserine lactone (Garlic extract)	Transcriptional regulators LuxR and LasR	*P. aeruginosa*	Decreased elaboration of virulence factors and reduced production of QS signals	[23–25]
Ethyl acetate fraction of *Cocculus trilobus*	Sortase	Gram positives bacteria	Exerted anti-adhesin effects at the adhesion stage of biofilm formation	[26]
Polyphenols (Cranberry)	Glucan-binding proteins, enzymes involved in biofilm formation	Cariogenic and periodontopathogenic bacteria	Affected the destruction of the extracellular matrix, carbohydrate production, bacterial hydrophobicity, proteolytic activities and coaggregation which involved in biofilm formation	[27–31]
Patriniae	Biofilm-associated genes	*P. aeruginosa*	Inhibited biofilm formation and reduced exopolysaccharide production	[32]
Ginkgolic acids	Curli genes and prophage genes	*E. coli* O157:H7	Inhibited biofilm formation on the surfaces of glass, polystyrene and nylon membranes	[33, 34]
Cinnamaldehyde	DNA-binding ability of LuxR	*E. coli* and *Vibrio* spp.	Affected biofilm formation and structure, the swimming motility, stress response and virulence	[35, 36]
Phloretin	Toxin genes (hlyE and stx(2)), autoinducer-2 importer genes (lsrACDBF), curli genes (csgA and csgB), and prophage genes in *E. coli* O157:H7	*E. coli* O157:H7	Reduced biofilm formation and fimbria production	[37]
Phloretin	Efflux protein genes	*S. aureus* RN4220 and SA1199B	Anti-biofilm formation at low concentration (1–256 μg/ml)	[38]
Isolimonic acid	luxO and AI-3/epinephrine activated cell–cell signaling pathway	*Vibrio harveyi*	Interfered with cell–cell signaling and biofilm formation	[39, 40]
Hordenine	QS-related genes	*P. aeruginosa*	Blocked QS-controlled phenotypes like biofilm formation and reduced virulence factors	[41, 42]
Quercetin	SrtA	*Streptococcus pneumoniae*	Blocked function of SrtA, affect sialic acid production and impair biofilm formation	[49]
Quercetin	LasI, LasR, RhlI and RhlR	*P. aeruginosa*	Inhibited biofilm formation and production of virulence factors	[44–48]
Quercetin	pH	*S. mutans*	Disrupted the pH in biofilm	[50]
Quercetin	Glycolytic, protein translation-elongation and protein folding pathways	*Enterococcus faecalis*	Blocked glycolytic, protein translation-elongation and protein folding pathways	[51]
Methanolic fraction of Z*ingiber officinale*	The virulence genes, F-ATPase activity, surface protein antigen SpaP	*S. mutans*	Inhibition of surface protein antigen SpaP and inhibitory effect on cell-surface hydrophobicity index of *S. mutans*	[53]
Ethanolic extract of *P. betle* leaf (PbLE)	Pyocyanin	*P. aeruginosa strain* PAO1	Inhibition of Pyocyanin production and reduction of swarming, swimming, and twitching ability of the bacteria by PbLE extract	[54]
*Bergenia crassifolia* (L.) leaf extract	Gtfs, EPSs	*S. mutans*	Decreased the adherence property of *S. mutans* through inhibiting Gtfs to synthesize EPSs	[55]
Ethanol extract from *Rhodomyrtus tomentosa*	Not investigated	*S. aureus, Staphylococcus epidermidis*	Inhibited staphylococcal biofilm formation and killed mature biofilm	[56]
Extract of *Hymenocallis littoralis* leaves	Adhesin proteins, SrtA and Als3	*S. aureus* NCIM 2654 *and C. albicans* NCIM 3466	Antimicrobial, anti-biofilm formation and antioxidant activities	[57]
Polyphenolic extract (Epigallocatechin-3-gallate) from *Camellia sinesis*	Not investigated	Stenotrophomonas maltophilia (sm) isolated from cystic fibrosis (CF)	Reduced bacterial cell viability in biofilm in vitro and significantly reduced Sm bacterial counts in an acute infection model with wild type and CF mice	[58]
Polyphenolic extract from *Rosa rugose tea*	QS-controlled violacein factors	*Chromobacterium violaceum* 026*, E. coli* K-12 and *P. aeruginosa* PAO1	Inhibited swarming motility and biofilm formation	[59]
Erianin	SrtA	*S. aureus*	Downregulated SrtA, thereby inhibited cell adhesion	[60]
Isovitexin	SpA	USA300	Reduced SpA and inhibited biofilm formation	[61]
Parthenolide	*LasI*, *RhlI*, *LasR*, *RhlR*, and extracellular polymeric substance	*P. aeruginosa* PAO1	Inhibited QS related genes expression including *LasI/LasR* and *RhlI/RhlR* and downregulated extracellular polymeric substance	[62]
Extract of *Chamaemelum nobile* flowers	Not investigated	*P. aeruginosa* PAO1 and strains isolated from patients	Inhibition of bacteria swarming and biofilm formation	[76]
Wheat-bran	AHL	*S. aureus*	Inhibition of QS and biofilm formation through downregulating AHLs level	[77]

QS, quorum sensing; SrtA, sortase A; SpA, Staphylococcal protein A; AHL, autoinducer acylhomoserine lactones

### Garlic

Garlic is considered as a rich source of many compounds with antimicrobial effects. It has been shown inhibitory effects on QS by garlic extract. In this regard, Bjarnsholt et al. found that garlic extract rendered *Pseudomonas aeruginosa* sensitive to tobramycin, respiratory burst and phagocytosis by polymorphonuclear leukocytes (PMNs) in a mouse pulmonary infection model [23]. Garlic was also found to decrease the elaboration of virulence factors and reduce production of QS signals in *P. aeruginosa* in a mouse UTI model [24]. Persson et al. found that garlic extracts showed inhibitory effects on biofilm formation against six clinical bacterial isolates. Furthermore, rational design and biological screening of all compounds from garlic also have been performed, resulting in the identification of a potent QS inhibitor *N*-(heptylsulfanylacetyl)-l-homoserine lactone. This component was demonstrated to interrupt QS signaling by competitively inhibiting transcriptional regulators LuxR and LasR [25].

### Ethyl acetate fraction of *Cocculus trilobus*

Kim SW and his colleagues reported that medicinal plant extracts from *C. trilobus* and *Coptis chinensis* could block the adherence of bacteria to surfaces with coated fibronectin. They exerted anti-adhesin effects at the adhesion stage of biofilm formation by suppressing the activity of a membrane enzyme named sortase which catalyzed the covalent anchoring of surface proteins to peptidoglycan in Gram-positives bacteria. Ethyl acetate fraction and water fraction were of these two plants was screened and ethyl acetate fraction of *C. trilobus* exhibited highest activity to suppress bacteria adhesin through targeting sortase [26].

### Cranberry polyphenols

Cranberry fruit is a rich source for polyphenols. Studies have reported that a non-dialysable cranberry fraction enriched in high molecular weight polyphenols inhibits biofilm formation and prevents the attachment and colonization of human pathogens, especially cariogenic and period onto pathogenic bacteria, to host tissues [27–30]. Moreover, Cranberry components affected Glucan-binding proteins, the activity of enzymes that cause the destruction of the ECM, carbohydrate production, bacterial hydrophobicity, proteolytic activities and coaggregation which involved in biofilm formation. The above-listed potential benefits of cranberry components suggest that especially those with high molecular weight polyphenols could serve as bioactive molecules with promising properties for the prevention and/or treatment of oral diseases, including dental caries and periodontitis [31].

### *Herba patriniae* extract

Fu et al. constructed a luxCDABE-based reporter system to detect the expression of six key biofilm-associated genes in *P. aeruginosa*. Then, 36 herb extracts were screened for inhibitory properties against those genes by this system. The results indicated that the extract from *Herba patriniae* displayed significant inhibitory effect on most of these biofilm-associated genes, which was in coincidence with a reduction in the biofilm formation and interference in the structure of the mature biofilms of *P. aeruginosa*. Moreover, *H. patriniae* extract reduced exopolysaccharide production in *P. aeruginosa*. These results revealed a potential candidate for exploration of new drugs against *P. aeruginosa* biofilm-associated infections [32].

### *Ginkgo biloba* extract

*Ginkgo biloba* extract was reported to significantly inhibit *Escherichia coli* O157:H7 biofilm formation on the surfaces of glass, polystyrene and nylon membranes at 100 µg/ml, without affecting bacterial growth. The mechanisms of inhibitory effects revealed that ginkgolic acid repressed curli genes and prophage genes in *E. coli* O157:H7, which were in-line with reduced fimbriae production and biofilm reductions [33, 34]. In another study, cinnamaldehyde was reported to affect biofilm formation and structure, and inhibited the swimming motility of *E. coli* [35]. Brackman et al. found that cinnamaldehyde and cinnamaldehyde derivatives interfere with biofilm formation, stress response and virulence of *Vibrio* spp. The mechanism of QS inhibition revealed an interference with AI-2 based QS in various *Vibrio* spp. by decreasing the DNA-binding ability of LuxR [36].

### Phloretin

Phloretin, as an antioxidant, is abundant in apples. Lee et al. found that it markedly reduced biofilm formation and fimbria production in *E. coli* O157:H7 strain without affecting the growth of planktonic cells. Phloretin also prevented *E. coli* O157:H7 attachment to human colon epithelial cells and suppressed the tumor necrosis factor alpha-induced inflammatory response. The mechanism of inhibitory effects revealed that phloretin repressed toxin genes (hlyE and stx(2)), autoinducer-2 importer genes (lsrACDBF), curli genes (csgA and csgB), and prophage genes in *E. coli* O157:H7 biofilm cells. This study suggested that phloretin also acts as an inhibitor of biofilm formation as well as an anti-inflammatory agent in inflammatory diseases [37]. Besides, Phloretin exhibited anti-biofilm formation of *S. aureus* RN4220 and SA1199B at low concentration with inhibitory efficiency up to 70% [38], which might function through targeting efflux proteins.

### Limonoids

Citrus limonoids are unique secondary metabolites for a triterpenoid. The purified limonoids present their ability to interfere with cell–cell signaling and biofilm formation in *Vibrio harveyi*, which seems to stem from the modulation of luxO expression, but not luxR promoter activity. Isolimonic acid and ichangin are potent modulators of bacterial cell–cell signaling [39]. The mechanism of the inhibitory effect of isolimonic acid, revealed that isolimonic acid and ichangin are potent inhibitors of biofilm and the type III secretion system. Furthermore, isolimonic acid appears to interfere with AI-3/epinephrine activated cell–cell signaling pathway in QseBC and QseA dependent fashion [40]. Zhou et al. firstly reported that hordenine showed a concentration-dependent reduction in signal molecule production and block QS-controlled phenotypes like biofilm formation in foodborne pathogen *P. aeruginosa.*

### Hordenine

Furthermore, hordenine effectively reduced virulence factors and QS-related gene expression of *P. aeruginosa* PAO1 [41, 42]. It is indicated that the anti-QS potential of hordenine act as a competitive inhibitor for signaling molecules and a novel QS-based agent to defend against foodborne pathogens [41]. Nanoparticles (NPs) like AuNPs was also developed to conjugate with hordenine and hordenine-AuNPs exhibited enhanced anti-biofilm properties on *P. aeruginosa* PAO1 [43], suggesting nanoparticles-delivered natural compounds could be effectively use in biofilm-based microbial infection.

### Quercetin

Quercetin, which exists in many fruits, vegetables and grains, is a plant polyphenol. It was reported to significantly inhibit biofilm formation and production of virulence factors including pyocyanin, protease and elastase at a lower concentration compared with that for most previously reported plant extracts and substances [44–47]. Further investigation of the transcriptional changes associated with QS found that the expression levels of LasI, LasR, RhlI and RhlR involved in QS signaling were significantly reduced [48]. Quercetin appeared to be an effective inhibitor of biofilm formation and virulence factors in *P. aeruginosa*. It was also identified as an effective inhibitor of SrtA, which could significantly impair biofilm formation of *Streptococcus pneumoniae* through suppressing sialic acid expression [49]. The anti-biofilm activities of quercetin in biofilm formation and biofilm-related infections were also investigated in *Streptococcus mutans* and *Enterococcus faecalis*, and results indicated the potential of quercetin in application for antimicrobial infection and anti-caries therapies for human health [50, 51]. Furthermore, nanoparticles decorated quercetin and quercetin conjugated microparticles showed more effective anti-biofilm activities [52], opening up a novel approach to develop therapeutic agent in prevention of microbial infections.

### Others

Other natural products or components such as polyphenolic extract from *Rosa rugose tea*, methanolic fraction of *Zingiber officinale* or kinds of leaves extract, were also demonstrated to display inhibitory effect on QS and biofilm development [53–55]. It was reported that ethanol extract from *Rhodomyrtus tomentosa* (Aiton) Hassk. leaf exhibited enhanced inhibitory effect on biofilm formation of *Staphylococcus aureus* than antimicrobial agent vancomycin [56]. Leaves extract of *Hymenocallis littoralis* contained multiple bioactive constituents (4-methylesculetin, methylisoeugenol, Quercetin 5,7,3′,4′-tetramethyl ether 3-rutinoside, phenols and flavonoids, etc.), which showed promising antimicrobial properties against pathogenic microorganisms and biofilm formation [57]. Green tea extract (epigallocatechin-3-gallate, EGCG) from *Camellia sinensis* could not only suppress *Stenotrophomonas maltophilia* biofilm formation both in vitro but also inhibit microbial infection in lung of C57BL/6 and *Cftr* mutant mice [58]. Meanwhile, polyphenolic extract from *Rosa rugose tea* also have anti-swarming activity on biofilm formation of *Chromobacterium violaceum* 026*, Escherichia coli* K-12 and *P. aeruginosa* PAO1 through targeting QS-related violacein factors [59]. Recently, emerging evidences also indicated that natural products such as erianin (from *Dendrobium chrysotoxum*), isovitexin and parthenolide exhibited inhibitory effect on cell adhesion, binding activity of fibronectin and QS factors respectively through targeting SrtA or downregulation of surface protein staphylococcal protein A (SpA) or blocking *P. aeruginosa* associated virulence factors, thereby impairing microbial biofilm formation [60–62].

The field of natural anti-biofilm agents screening has consistently widened. Besides those anti-biofilm agents listed above, other ones extracted from herbs [63, 64], India medicinal plants [65], natural phenolic compounds [66], green tea and mouthwash [67], mushroom [68], licorice root [69], Polish Propolis [70], *Allium sativum* [71], *Psidium cattleianum* leaf [72], *Solidago virgaurea* [73], *Roselle calyx* [74], *Juglans regia* L. [75], also emerged in these last years. These studies mainly performed pilot studies like the evaluation of the anti-biofilm activity against common bacteria and biofilm-related biological effects, which basically implied their potential in anti-biofilm therapy in infectious diseases. However, many properties of those novel anti-biofilm agents were still not well characterized, such as the molecular mechanisms involved in controlling and perturbation of QS signaling, or the molecular structures of the bioactive ingredients which exerted the anti-biofilm activity, etc. It was found that extract from the flowers of *Chamaemelum nobile* inhibited QS activity and biofilm development to combat *P. aeruginosa* strain PAO1 and strains isolated from infected patients [76]. However, the molecular mechanism underlying anti-biofilm action as well as the functional constitutes need to be further investigated and identified. The Wheat-bran also potentiated anti-biofilm activity and was demonstrated to disrupt QS system by downregulating QS signal molecules acyl-homoserine lactones (AHL) [77]. Further studies were still needed to identify the potential active components in wheat-bran as well as other effective extract from natural plants, aiming to derive new effective compound to control biofilm formation and pathogenic bacterial infection.

## Separation and extraction of bioactive anti-biofilm components from plants

Although lots of herbs extract have been already demonstrated exhibiting anti-biofilm effects, the bioactive molecules or components are still unknown and need to be further investigated. Thus, the separation and extraction of effective anti-biofilm components are important. Over the past decade, techniques like chromatographic separation and structure-based virtual screening (SB-VS) have been extensively screened to identify bioactive ingredients acting as anti-biofilm agents from plants, laying solid foundation of excavating novel molecules for biofilm control and bacterial infection. We also summarized the techniques used in the separation and identification of bioactive ingredients in plants extract (Table 2). Below is the brief description of these techniques.

**Table 2:** Separation and extraction of bioactive anti-biofilm components from plants

Plant	Bioactive components	Methodology	References
Assam tea	Galloylated catechins	HPLC	[78]
Several common food products and plants	Iberin	LC-DAD-MS and NMR spectroscopy	[79]
Coconut husk extract	One bioactive OH-group-containing compound	TLC, HPLC and FT-IR analysis	[80]
12 herbs in Thailand	4-Chromanol	GC–MS analysis, TLC fingerprinting and TLC-bioautography	[81]
*Schinus terebinthifolius*	Phenolic compounds, anthraquinones, terpenoids, and alkaloids	TLC analysis	[82]
Pomegranate extract	Ellagic acid	HP-TLC analysis	[83]
Medicinal plants		UPLC analysis	[84]
1920 natural compounds/drugs	Rosmarinic acid, naringin, chlorogenic acid, morin and mangiferin	SB-VS against LasR and RhlR receptor	[85]
3040 natural compounds and their derivatives.	5-Imino-4,6-dihydro-3H-1,2,3-triazolo[5,4-d]pyrimidin-7-one	SB-VS against the QS receptor LasR	[46]
51 bioactive components from Traditional Chinese Medicines (TCMs)	Baicalein	SB-VS against transcriptional activator protein TraR	[86]
46 bioactive components from TCMs	Emodin	SB-VS against transcriptional activator protein TraR	[87]
Natural and synthetic compound libraries	4-NPO	Screening systems named QSI selectors	[88]
Five commercial tea extracts	Polymeric and monomeric tea phenolics	Phytochemical screening	[89]

### Chromatographic separation

Kawarai et al. reported that tea could inhibit the attachment of *Streptococcus mutans* to surfaces and subsequent biofilm formation. Assam tea presents more potent anti-biofilm activity against *S. mutans* than green tea. Ultrafiltration with centrifugal filter devices and high-performance liquid chromatography (HPLC) were utilized for the purification and identification of QSI in Assam tea. A substance in Assam tea, with molecular weight less than 10 kDa, had a high concentration of galloylated catechins and a stronger anti-biofilm activity than green tea. However, polysaccharides such as pectin, > 10 kDa in mass from green tea was found to enhance biofilm formation [78].

In another study, several common food products and plants were extracted and screened to isolate the unknown components with active QSI activity. Iberin, as an isothiocyanate produced by the Brassicaceae family, was identified by liquid chromatography-diode array detector-mass spectrometry (LC-DAD-MS) and nuclear magnetic resonance (NMR) spectroscopy. Suppression of QS-regulated genes was further demonstrated by Real-time PCR (RT-PCR) and DNA microarray assays [79].

The effects of coconut husk extract (CHE) on extracellular polymeric substance (EPS) production, hydrophobicity and adhesion ability involved in biofilm formation in *Pseudomonas* spp., *Alteromonas* spp. and *Gallionella* spp. were tested. CHE was found to affect the EPS production and hydrophobicity of the bacterial cells, as well as exert antibacterial activity against all the bacterial strains. Analyzing by thin-layer chromatography (TLC), HPLC and Fourier transform infrared (FT-IR) assays, one bioactive OH-group-containing compound of CHE was found in the extract [80].

Teanpaisan and his colleagues tested the anti-biofilm activity of 12 herbs in Thailand. *Piper betle* acted as the most potent anti-biofilm agent in 12 Thai traditional herbs against oral pathogens. It exerted dual actions including preventing and eradicating the biofilm. The bioactive compounds characterized by GC–MS analysis, TLC fingerprinting and TLC-bioautography was 4-chromanol, the major constituent of *Piper betle* extract, which was demonstrated to be responded for antibacterial and anti-biofilm against oral pathogens [81]. *Schinus terebinthifolius Raddi*, from the Anacardiaceae family, is a popular plant used in folk medicine for treatment of several health disorders in Brazil, which is found to exert antimicrobial, anti-inflammatory and antiulcer properties. Barbieri et al. found that *S. terebinthifolius* efficiently inhibited the biofilm formation and adherence of *Candida albicans.* Results in TLC analysis showed the presence of several bioactive compounds in *S. terebinthifolius* extracts, including phenolic compounds, anthraquinones, terpenoids, and alkaloids. The findings indicated the potential applications of natural products in the therapeutic prevention of oral diseases associated with oral biofilms [82].

Pomegranate is a common fruit and is also utilized traditionally to treat various ailments. A methanolic extract of pomegranate was used to detect the anti-biofilm activity of against bacterial and fungal pathogens. The methanolic fraction of pomegranate was found to inhibit the biofilms formation produced by several bacteria including *S. aureus,* methicillin-resistant *S. aureus*, *E. coli*, and *Candida albicans.* Moreover, pomegranate extract also disrupted germ tube formation with respect to virulence in *C. albicans*. High-pressure thin layer chromatography (HP-TLC) was performed in order to determine the prime component of pomegranate extract. It revealed the presence of the ellagic acid as the major component [83]. Medicinal plants are an important source and used traditionally for the therapeutic remedies of infectious diseases. A study aimed to determine the influence of some plant extracts including *Betula pendula, Equisetum arvense, Herniaria glabra, Galium odoratum, Urtica dioica*, and *Vaccinium vitisidaea* on virulence factors expression and biofilm formation of the uropathogenic *E. coli* rods. Compounds identification was performed on an Acquity ultra-performance liquid chromatography (UPLC) system coupled with a quadrupole-time of flight (Q-TOF) MS instrument (UPLC-Q-TOF–MS). All the extracts of those medical plants showed the anti-biofilm activity. Moreover, some extracts presented their inhibitory effects on growth, surface hydrophobicity and the motility of the *E. coli* rods [84].

### Structure-based virtual screening (SB-VS)

Many studies have pointed out QS system as a new, promising target for antimicrobial drugs. Against targets in QS signaling pathways, structure-based virtual screening (SB-VS) and in silico docking analysis were utilized to search for putative QSI of *P. aeruginosa*. Five top-ranking compounds were screened from about 2000 natural compounds against LasR and RhlR receptor in *P. aeruginosa*. The pharmacological effects of five top-ranking compounds, namely rosmarinic acid, naringin, chlorogenic acid, morin and mangiferin were subjected to in vitro bioassays against strain PAO1 and two antibiotic-resistant clinical isolates. Most of these compounds significantly inhibit the production of virulence factor and potentially inhibited the biofilm related behaviors [85]. In a separated study, SB-VS approach was used to screen novel QSI candidates from 3040 natural compounds and their derivatives. Using the QS receptor LasR as a target, 22 compounds were obtained based on docking scores and molecular masses and further investigations were performed to determine their efficacies as QSI. Using a live reporter assay for QS, 5 compounds were demonstrated to be able to suppress QS-regulated gene expression in *P. aeruginosa* in a dose-dependent manner and obviously regulate 46 proteins (19 were upregulated; 27 were downregulated) including several quorum-sensing-regulated virulence factors, such as protease IV, chitinase, and pyoverdine synthetases in *P. aeruginosa* PAO1 [46].

Traditional Chinese Medicines (TCMs) provided a huge database for QSI screening. Using computer-based virtual screening, 51 bioactive components of Traditional Chinese Medicines with antibacterial activity were screening for QSIs of *P. aeruginosa*. Baicalein inhibits biofilm formation of *P. aeruginosa* and does not inhibit the growth of *P. aeruginosa*. It promoted the proteolysis of the signal receptor TraR protein in *E. coli* [86]. In another virtual screening based on molecular docking, six compounds were found in 46 bioactive components from TCMs as putative QSIs. Three compounds exerted anti-biofilm effects in *P. aeruginosa* and *Stenotrophomonas maltophilia*. Moreover, emodin significantly inhibited biofilm formation and also promoted proteolysis of the signal receptor TraR in QS in *E. coli*. Emodin increased the activity of ampicillin against *P. aeruginosa* [87]. Therefore, the components from TCMs like emodin and baicalein could be developed as quorum sensing inhibitors with the novel target for anti-virulence and antibacterial therapy.

These interaction studies demonstrate the utility of SB-VS for the discovery of target-specific QSIs and provided potential candidates to inhibit the QS-controlled biofilm formation and virulence factors production.

### Others

Besides the two methodologies listed above, other screening techniques were also employed for the discovery of anti-biofilm agents. Rasmussen et al. constructed a collection of screening systems named QSI selectors and then selected novel QSIs among natural and synthetic compound libraries. As a result, garlic extract and 4-nitro-pyridine-*N*-oxide (4-NPO) were identified by the QSI selectors as the most bioactive components. Furthermore, specificity for QS-controlled virulence genes and pharmacological effects were demonstrated by GeneChip-based transcriptome analysis and in a *Caenorhabditis elegans* pathogenesis model [88].

Five commercial tea extracts were screened for their inhibitory effects on attachment and biofilm formation by two strains of *S. mutans*. Furthermore, using scanning electron microscopy (SEM) and phytochemical screening. The results indicated that extracts of oolong tea and pu-erh tea most effectively suppressed attachment and biofilm formation of *S. mutans*, respectively. The inhibitory effect of tea extracts on cell attachment and biofilm formation in the current study may be induced by large molecules in the extracts or the synergistic effect of polymeric and monomeric tea phenolics. This study indicated potential mechanisms like modification of cell surface properties, blocking of the activity of proteins, alterations in the structures used by the bacteria to interact with surfaces, which could explain the inhibitory effects of tea components on the attachment and subsequent biofilm formation of *S. mutans* [89].

## Natural anti-biofilm agents under clinical evaluation

Up to now, no anti-biofilm agents for infectious diseases have been approved by U.S. Food and Drug Administration yet. However, some natural anti-biofilm agents have been systemically investigated in clinical trials, exhibiting a promising perspective. Various clinical ongoing phase I, II III and IV trials of anti-biofilm agents as a single anti-biofilm agent have been performed in patients (reported in http://clinicaltrials.gov/), but some completed clinical trials did not post the results yet. Resources with the outcome reported from http://clinicaltrials.gov/are listed in Table 3.

**Table 3:** Natural anti-biofilm agents under clinical evaluation (http://clinicaltrials.gov/)

Condition	Status	Intervention	Phase	Year
Biofilms Essential oils Periodontitis	Not yet recruiting	Drug: Essential oils Drug: Essential oils without alcohol Drug: Sterile water	IV	2016
Biofilms Substantivity Essential oils	Not yet recruiting	Drug: Essential oils Drug: Essential oils without mouthwash Drug: Sterile water	IV	2016
Oral biofilm Dental plaque Periodontitis	Recruiting	Drug: Essential oils Drug: Alcohol free essential oils Other: Water	IV	2017
Streptococcal infections Saliva altered	Completed	Other: Propolis varnish	I and II	2015
Gingivitis	Completed	Dietary supplement: Black tea Dietary supplement: Green tea Drug: 0.12% chlorhexidine mouthwash	III	2015
Oral biofilm Mouthwash Periodontitis	Completed	Drug: Essential oils Drug: 0.2% chlorhexidine Drug: Sterile water	IV	2013
Dental biofilm pH	Completed	Other: G1, G2 and G3 Other: G4 and G5	II	2012
Gingivitis	Completed	Drug: *Punica granatum* Linn. Drug: chlorhexidine	Not applicable	2012
Prostheses-related infections	Completed	Other: Physiological solution Other: Sodium hypochlorite Other: Alkaline peroxide Other: Castor bean solution	Not applicable	2011

There were some clinical trials related to natural products achieving favorable outcomes in anti-microbial effect and anti-biofilm activities, which are listed in Table 4. Most of these clinical trials focus on the anti-bacterial and anti-biofilm effect on patients with denture transplantation or dental inflammation or oral disease by using natural products as a component of mouthwash or dentifrice. There were clinical trials by using 10% *Ricinus communis* to treat denture wearers with stomatitis and results indicated favorable antibacterial efficacy of *Ricinus communis* against *S. mutans* and *Candida* spp. [90, 91]. There were some clinical trials indicated that mouthrinses containing herb extracts such as combination of green tea and *Salvadora persica* L. *aqueous* extracts or *Matricaria chamomilla* L. (MTC) extract had significantly positive effect on dental biofilm control and significantly decrease the plaque colonization [92, 93]. Mouthwash containing essential oil might also be more effective in killing dental bacteria. A randomized-controlled clinical trials involving patients with moderate chronic periodontitis indicated that combined using mouthwash with essential oil from *Cymbopogon flexuosus*, *Thymus zygis* and *Rosmarinus officinalis* had anti-biofilm potential in the subgingiva in patients with good tolerability [94]. As indicated in a randomized double-blind trail, mouthrinses consist of Lemongrass oil reduced oral malodour for 8-day treatment, which might due to its selectively anti-microbial effect against *Aggregatibacter actinomycetemcomitans* ATCC43718 and *Porphyromonas gingivalis* W50 [95]. Dentifrice containing natural components such as essential oil of *Melaleuca alternifolia* or vegetable oil also exhibited enhanced anti-bacterial and improved biofilm control effect in patients with orthodontic treatment or with caries when compared with commercial dentifrice [96, 97]. There were also some clinical trials related to the treatment of natural anti-biofilm products on urinary tract infection (UIT). Oral Cranberry extract (proanthocyanidin-A, PAC-A) seemed play an important role in anti-bacterial infection in prevention of UIT [98] and a formula consisting of cranberry extract, solidago, orthosiphon and birch (CISTIMEV PLUS^®^) evidently decreased bacterial accumulation in patients with indwelling urinary catheters in clinical [99]. On the other hand, it seemed that some clinicals could not achieve promising outcomes, which might be due to the limited samples size or different treatment time [100]. Moreover, most of these clinical trials resulted in positive effect of natural ingredients on reduction of bacterial survival and biofilm formation, suggesting good prospect in clinical application of natural anti-biofilm products. Overall, we found that the sample size was all ranged from 30 to 70 participates, indicating that more researches needed to be optimized and performed with enlarged sample size. Regarding that these trials mainly focusing denture or stomatology related clinical trials, it is promising to include more infected disease to do clinical evaluation on natural anti-biofilm agents.

**Table 4:** Natural anti-biofilm agents under clinical evaluation with outcomes

Studies	Condition	Intervention	Outcome	References
Randomized controlled clinical trials	Denture wearers with denture stomatitis (n = 64)	Control, 0.85% saline SH1, 0.25% sodium hypochlorite SH2, 0.5% sodium hypochlorite RC, 10% *Ricinus communis*	*Ricinus communis* showed antimicrobial activity against *S. mutans* and *Candida* spp.	[90]
A randomized crossover clinical trial	Denture wearers with denture stomatitis (n = 50)	Control, 0.85% saline SH1, 0.1% sodium hypochlorite SH2, 0.2% sodium hypochlorite RC, 8% *Ricinus communis*	*Ricinus communis* alleviated the symptom of denture stomatitis, however anti-biofilm effect was not evident	[91]
Randomized controlled trials	Dental plaque (n = 14)	Control, 0.12% chlorhexidine Test formulation containing 0.25 g/ml green tea and 7.82 g/ml *Salvadora persica L. aqueous* extracts Placebo mouthwashes	The test mouthwash significantly has positive effect on disrupting plaque colonization when compared with placebo and control group for short time treatment (24 h)	[92]
Randomized, double-blind controlled study	Patients undergoing orthodontic treatment with fixed appliances (n = 30)	C: Placebo T1: mouthwash containing 1% *Matricaria chamomilla* L. (MTC) extract T2: 0.12% chlorhexidine (CHX)	MIC could suppress biofilm development and gingival bleeding	[93]
Randomized controlled clinical trials	Patients with moderate chronic periodontitis after scaling and root planing (SRP) (n = 46)	Placebo mouthwashes (n = 23) Essential oil mouthwash consisting of essential oils (*Cymbopogon flexuosus*, *Thymus zygis* and *Rosmarinus officinalis*) (n = 23)	The combined use of a mouthwash containing essential oils following SRP was well tolerated and had anti-biofilm effect in the subgingival for 14-day treatment	[94]
Randomized double-blind clinical study	Oral malodour (n = 20)	*Lemongrass oil* (LG) mouthrinse	LG mouthwash showed selective anti-bacteria effect against *Aggregatibacter actinomycetemcomitans* ATCC43718 and *Porphyromonas gingivalis* W50 and could reduce oral malodour for 8-day treatment	[95]
Randomized controlled trials	Orthodontic patients (n = 34)	Melaleuca gel: Gel developed with the essential oil of *Melaleuca alternifolia* Colgate total	The melaleuca gel was more effective in decreasing the dental biofilm and the numbers of bacteria colonies	[96]
Randomized controlled trials	Caries and periodontal diseases (n = 30)	G1: A commercially available dentifrice G2: Dentifrice containing mineral oil (Nujol^®^) G3.: Dentifrice containing vegetable oil (Alpha Care^®^)	Both mineral oil and vegetable group exhibited improved biofilm control and could significantly decrease dental biofilm formation in clinical	[97]
Randomized controlled trials	Patients with subclinical or uncomplicated recurrent UTI (r-UTI) (n = 72)	Placebo (n = 36) *Cranberry* extract (PAC-A, proanthocyanidin-A) (n = 36)	The overall efficacy and tolerability of standardized cranberry extract containing (PAC-A) as a food supplement were superior to placebo in terms of reduced bacterial adhesion; biofilm development; urine pH reduction; and in preventing r-UTI (dysuria, bacteriuria and pyuria)	[98]
Single-blinded, randomized and controlled pilot study	Patients with indwelling urinary catheters (n = 83)	Control (n = 35) CISTIMEV PLUS^®^: *Solidago*, *orthosiphon*, *birch* and *cranberry* extracts (n = 48)	CISTIMEV PLUS^®^ significantly reduced microbial accumulation in patients	[99]
A pilot randomized controlled trial	*P. aeruginosa* related lung cystic fibrosis (n = 34)	Placebo group Garlic or olive oil treatment group	Both garlic and olive oil capsules were tolerated, but no significant effect was found in antibacterial activities	[100]

## Conclusions and perspectives

The biofilms are identified with increased resistance to antibiotics and antimicrobial agents, causing a troublesome burden on human health care. Treatment of biofilm-associated infections is currently a complicated challenge for clinicians and microbiologists. Novel antimicrobial strategies are urgent to be developed to transcend problems with antibiotic resistance in microbial infectious diseases.

As indicated in this review, nature resources offered a huge library for the screening of anti-biofilm agents. Plants and natural foods have been gaining increasing research focus on their health-promoting effects in recent years. Up to now, series of studies investigated inhibitory effects of natural products on bacterial biofilm formation and development, suggesting their potential as alternative agents for bacterial infections. According to the current findings, most of natural anti-biofilm agents showed encouraging preclinical data for anti-biofilm efficiency in various bacterial species. Their potential regulatory mechanism was mainly due to the suppression of each steps of biofilm formation or QS network inhibition. Screening anti-biofilm agents by chromatographic separation and other techniques in the last two decades opened the way to isolate effective components. However, many studies of plant-derived extracts with anti-biofilm activity do not identify the molecular structures of the bioactive molecules, indicating that more studies need to be performed. Moreover, it is encouraging that investigations on natural anti-biofilm agents based anti-infective therapy are undergoing for phase I–IV clinical trials. Ongoing clinical trials mainly focus on external use for oral biofilm produced in dental plaque, periodontitis and gingivitis. It is worthy to evaluate the effectiveness and tolerance of natural products in clinical patients with deep-located biofilm-related infection, e.g. in the viscera, tissues or other organs inside. Meanwhile, improved specificity, safety, efficiency alone or in combination with other antibiotics are also required for the development and evaluation of natural anti-biofilm agents in clinical application, which would produce a great impact on the control of bacterial infectious diseases and benefit a lot for heathy care throughout the world.

## Abbreviations

AHL: acylhomoserine lactone
AIP: autoinducer peptide
BAP: biofilm associated protein
CHE: coconut husk extract
ECM: extracellular matrix
EGCG: epigallocatechin-3-gallate
FT-IR: Fourier transform infrared
HPLC: high-performance liquid chromatography
LC-DAD-MS: liquid chromatography-diode array detector-mass spectrometry
MTC: *Matricaria chamomilla* L.
NP: nanoparticle
NMR: nuclear magnetic resonance
PCL: programmed cell lysis
PAC-A: proanthocyanidin-A
PMN: polymorphonuclear leukocyte
QS: quorum sensing
QSI: QS inhibitor
SB-VS: structure-based virtual screening
SEM: scanning electron microscopy
SrtA: sortase A
Spa: surface protein antigen
SpA: Staphylococcal protein A
TCM: Traditional Chinese Medicines
TLC: thin-layer chromatography
UPLC: ultra-performance liquid chromatography
4-NPO: 4-nitro-pyridine-*N*-oxide
